# Close of the Volume

**Published:** 1857-12

**Authors:** 


### EDITORIAL.

### CLOSE OF THE VOLUME.

The present number completes the sixth volume of the new series of the *North-Western Medical and Surgical Journal*, and in it will be found the title-page and index to the volume. The large supply of original matter has crowded out all the selections and a part of the book notices prepared for the present number. Having completed arrangements for such an amount of editorial assistance as will insure an efficient superintendence of every department of the *Journal*, we are confidently expecting to make the forthcoming volume better than any of its predecessors. We earnestly invite contributions from practitioners and societies throughout the North-Western States, and pledge to them an active co-operation in all that relates to the advancement and usefulness of the profession. With the commencement of the new volume we shall adopt a new name. The present long and awkward title was chosen when Chicago was really close upon the north-western border of our magnificent Union; but at present it would be much more applicable to a journal published in Oregon or Washington Territory. Hence, the January number will be issued under the title of the "CHICAGO MEDICAL JOURNAL."

We have a large subscription list, and if all those whose names are on it would promptly pay up, we would immediately enlarge the *Journal*, by adding sixteen pages more of reading matter to each number. All remittances and communications relating to the business of the *Journal* should be addressed, as heretofore, to the senior editor, Dr. N. S. Davis, Chicago, Ill.

